# Guidance on left bundle branch pacing using continuous pacing technique and changes in lead V1 characteristics under real-time monitoring

**DOI:** 10.3389/fcvm.2023.1195509

**Published:** 2023-09-29

**Authors:** Nan Zheng, Longfu Jiang, Jiabo Shen, Jinyan Zhong

**Affiliations:** Department of Cardiovascular Medicine, Ningbo NO.2 Hospital, Ningbo, China

**Keywords:** left bundle branch pacing, left bundle branch capture, physiological pacing, left ventricular septal capture, electrocardiogram

## Abstract

**Background:**

The changes in the morphology and characteristics of the V1 leads during left bundle branch capturing still need to be fully understood.

**Objective:**

This study aims to provide some suggestions about the LBB capture process through the morphology and characteristics of the V1 lead.

**Method:**

LBBP using the continuous pacing and morphology monitoring technique during screw-in using a revolving connector (John Jiang's connecting cable). The morphology and features of V1 leads are recorded by continuous monitoring technology.

**Results:**

The most common morphology in the LVSP stage is QR, while in the NS-LBBP (low output) stage and the NS-LBBP (lower output) stage, it is rSR. In the S-LBBP stage, it is rsR. The predominant morphology is with r/R waves in S-LBBP, which includes variations like rSR, rsR, rSr, rsr, rR, rs, rS, and R type, making up 96.7% of the total. The r waves in lead V1 are associated with agitated myocardium conducted from the left bundle branch.

**Conclusion:**

The initial r-wave in lead V1 may be a marker during the follow-up of patients with selective LBB capture.

## 1. Introduction

The only effective treatment for symptomatic bradycardia without reversible causes is cardiac pacing. Left bundle branch (LBB) pacing (LBBP) provides a low and stable pacing threshold and a short R-wave peak time (RWPT) as an alternative to His Bundle Pacing (1, 2).

During LBB capture, electrocardiogram (ECG) features are exceptionally critical, especially changes in the morphology and amplitude of lead V1. Because the primary polarization vector of the QRS is from right to left and deviated from V1. The V1 leads provide a more precise reflection of the left and right ventricular agonistic sequences (3). In particular, the interval between left and right ventricular excitation in the right bundle branch block is elongated, showing an anterior-posterior sequence.

However, the continuous recording has yet to be applied to the techniques reported about left bundle branch pacing. This has resulted in a current lack of ECG changes at important nodes and an omission of the different phases of V1 lead capture in the left bundle branch.

Previous knowledge of V1 lead morphology during left bundle branch capture still needs to be completed. In other studies of capture criteria, the morphology of the V1 leads is more generalized to present the morphology of the right bundle branch block rather than a specific individual morphology (4). However, the V1 lead presents a right bundle branch block during left ventricular septal pacing.

Therefore, this article retrospectively summarizes the morphology of V1 leads in our center for selective left bundle branch pacing. We hope to establish a new marker from changes in V1 morphology that can give centers without continuous recording techniques some additional ideas and be able to monitor over the course of long-term follow-up.

## 2. Methods

### 2.1. Patients

Patients who underwent LBBP at the Chinese Academy of Sciences Ningbo Hwamei Hospital between May 2021 and June 2023 were screened in this study. Of the 160 patients, 8 (5%) were identified as non-selective left bundle branch pacing, and 152 (95%) met the selective left bundle branch pacing criteria. The present study is a retrospective analysis of these patients with selective left bundle branch pacing.

All pacemaker indications for patients included in the study were performed according to the latest guidelines (5–7). The study was guided by the Declaration of Helsinki and its subsequent amendments. The hospital's ethics review board approved the study protocol (SL-KYSB-NBEY-2021-079-01), and all patients signed written informed consent.

### 2.2. Left bundle branch capture

LBBP using the continuous pacing and morphology monitoring technique during screw-in using a revolving connector (John Jiang's connecting cable) have been described elsewhere (8–10).

**Figure 1:**
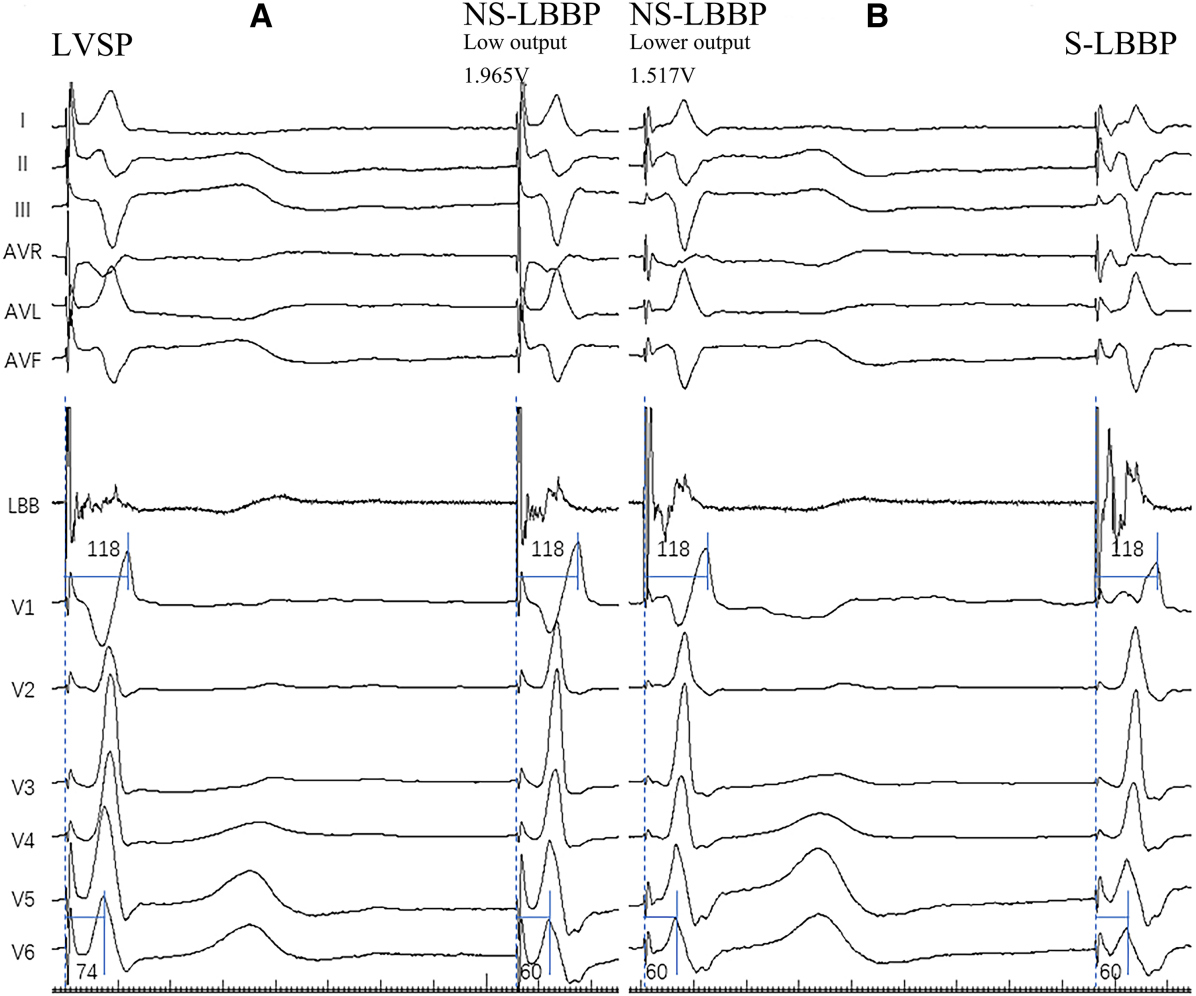
The four pacing modalities of the implantation. (**A**) Body surface 12-lead ECG and LBB potential in both LVSP and NS-LBBP (Low output) phases. Two of the nodes are adjacent to heartbeats. In the deeper septum and LV endocardium, when the stim-V6 RWPT is abruptly shortened, the pacing modality changes from LVSP to NS-LBBP. (**B**) Body surface 12-lead ECG and LBB potential in NS-LBBP (Lower output) and S-LBBP phases. RWPT-V6 is 60 ms at NS-LBBP (lower output) and 60 ms at S-LBBP. Two of the nodes are adjacent to heartbeats. At the LBB, the pacing modality transitions from NSLBBP to SLBBP when a discrete component appears on the intracardiac electrogram with fixed stim-V6RWPT.

The following steps perform the pacing of the left bundle branch. After the tricuspid valve angiogram identified the right screw-in point, the 3,830 electrodes were connected with John Jiang's connecting cable and screwed in at 2 V/0.5 ms with continuous pacing, using an Abbott EP-WorkNET digital electrophysiology system with the high-pass filter set to 200/500 HZ. After two adjacent heartbeats at 2 V/0.5 ms output with a significantly shorter V6 RWPT, pause the electrode screwing and quickly lower the output to observe whether the S-V separation is present, if the V6 RWPT is instead prolonged, keep the output low and continue screwing the electrode with caution until the S-V separation is present. We define the adjacent V6 RWPT as LVSP before shortening; “NS-LBBP (low output)” after shortening; “NS-LBBP (lower output)” before S-V separation, and S-V separation as S-LBBP (2, 10) (Figure 1).

### 2.3. Data acquisition

The patient's baseline data is registered by Z.N. on the information in the medical record system and confirmed with the patients. Diagnostic information is verified by a physician specializing in cardiology at the hospital, and critical tests and laboratory data are retained.

The implantation procedure was recorded on a digital electrophysiology system EP-WorkNET (Abbott Laboratories, Chicago, IL). The V1 morphology of 12 lead ECGs of the whole process was recorded and derived, the ECG morphology before and after the four stages was distinguished, and the amplitudes of the Q/S waves and R waves were measured. Simultaneously, a stimulus to V6 R-wave peak time (stim- V6 RWPT) measurements was performed using the EP system at 200 mm/s.

### 2.5. Statistical analysis of data

The normality distribution was first tested for the continuous variables, and the data conforming to the normal distribution were expressed as mean ± standard deviation. The paired samples *t*-test was used in the subsequent paired tests. Continuous variables that did not conform to a normal distribution were represented in quartile form, and the Wilcoxon rank sum test was used in the subsequent paired tests. Categorical variables are expressed as proportions. The chi-square test was used to test the variability of categorical variables across subgroups. *P* values less than 0.05 were considered statistically significant. Statistical analysis was performed using IBM SPSS Statistics for Macintosh (version 26.0, IBM Corporation, Armonk, NY).

## 3. Results

### 3.1. Baseline data

The statistics of the baseline data are shown in Table 1. Among the pacemaker indications, Atrioventricular Block and Sick Sinus Syndrome accounted for the most, 65% and 30%, respectively. 3% of these patients had heart failure with reduced ejection fraction, and 2 had heart failure with fast ventricular rate atrial fibrillation, for which pharmacologic therapy was unsatisfactory and AV node ablation with pacemaker implantation was performed. 2% of the patients had atrial fibrillation with slowed ventricular rate as the indication for pacemaker implantation (5–7). For the four stages of RWPT of V6 leads, the same analysis was performed, and the results showed that in the LVSP stage, the RWPT of V6 leads was 82 ms, the V6 RWPT of NS-LBBP (Low output) was 68 ms, the V6 RWPT of NS-LBBP (Lower output) was 68 ms, and the V6 RWPT of S-LBBP was 66.5 ms.

**Table 1:** Baseline. Output is the output voltage used during the hyperacute phase after electrode wire implantation. Threshold is the threshold on the posterior side of the stabilization period. The results show that a more significant proportion of male patients, 55%, were included among the patients. In addition, among male patients, the mean age was 76 and 73 for female patients. Among the pacemaker indications, Atrioventricular Block (AVB) and Sick Sinus Syndrome (SSS) accounted for the most, 65% and 30%, respectively. For the four stages of RWPT of V6 leads, the same analysis was performed, and the results showed that in the LVSP stage, the RWPT of V6 leads was 82 ms, the V6 RWPT of NS-LBBP (Low output) was 68 ms, the V6 RWPT of NS-LBBP (Lower output) was 68 ms, and the V6 RWPT of S-LBBP was 66.5 ms.

	Classification	mean ± sd or quartile
Age(years)	female	73 (66.00,79.00)
	male	76 (71.00,81.25)
Gender	female	69 (45%)
	male	83 (55%)
Diseases	atrial fibrillation with slowed ventricular rate	3 (2%)
	atrioventricular block	99 (65%)
	heart failure with reduced ejection fraction	5 (3%)
	sick sinus syndrome	45 (30%)
left ventricular end-diastolic dimension (mm)	female	49 (44.75,51.25)
	male	51 (45.00,54.00)
left ventricular ejection fraction (%)	female	63 (59.00,70.00)
	male	63 (58.00,68.25)
R-wave peak time -V6 (ms)	LVSP	82.00 (75.00,89.00)
	NS-LBBP (low output)	68.00 (63.00, 73.00)
	NS-LBBP (lower output)	68.00 (62.00, 72.00)
	S-LBBP	66.50 (62.00, 72.00)
Output(v)	LVSP	1.965 ± 0.422
	NS-LBBP (low output)	1.965 ± 0.422
	NS-LBBP (lower output)	1.488 ± 0.736
	S-LBBP	1.488 ± 0.736
Threshold(v/0.5 ms)	left ventricular septal	1.252 ± 0.883
	left bundle branch	0.779 ± 0.511

### 3.2. Morphology in the V1 lead

**Figure 2:**
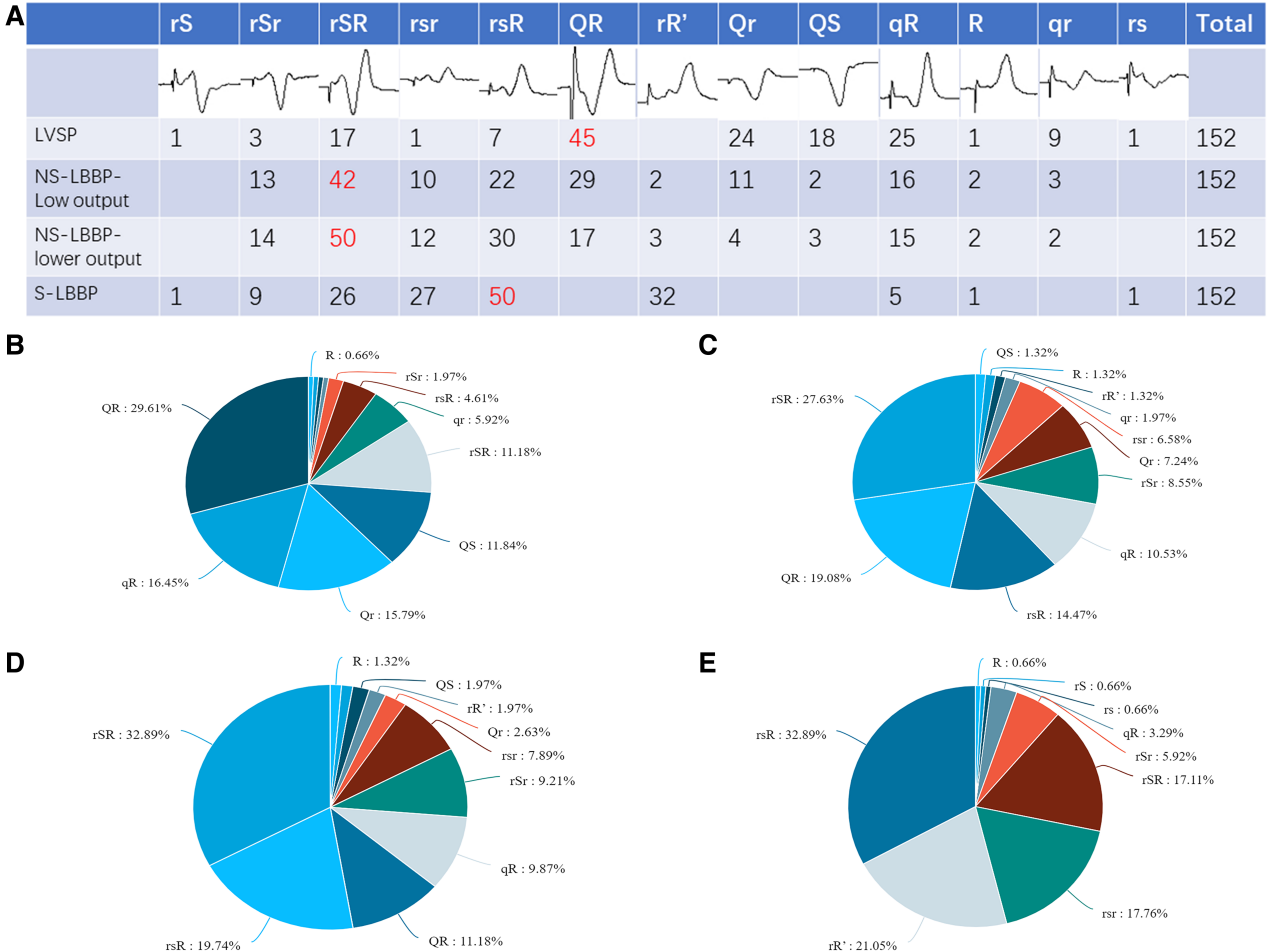
V1 morphology for the four key nodes. (**A**) Graphical and quantitative statistics of all morphology for the four nodes, with the most common morphology marked in red. (**B**) Proportion of V1 morphology at the LVSP stage. (**C**) Proportion of V1 morphology at the NS-LBBP (Low output) stage. (**D**) Proportion of V1 morphology at the NS-LBBP (Lower output) stage. (**E**) Proportion of V1 morphology at the S—LBBP stage.

Summarizing and statistics of QRS morphology in the V1 lead of the four nodes (LVSP, NS-LBBP (low output), NS-LBBP (lower output), S-LBBP) showed 13 different morphologies. These morphologies were: rS, rSr, rSR, QR, rR’, Qr, QS, qR, rsR, rsr, R, qr, rs. The statistics of V1 morphology for the four key nodes are shown in Figure 2.

Part B of Figure 2 shows that the most common morphology in the LVSP stage was QR, which accounted for 29.61% of the total, followed by qR, which occupied 16.45%. The two most common similar morphology accounted for about 46.06% of all patients. Similar morphology, such as QR, Qr, qR, qr, and QS, accounted for 79.61% of the total and most patients.

Part C of Figure 2 shows that the most common morphology in the NS-LBBP phase was rSR, which accounted for 27.63% of the total. Similar morphology such as rSR, rsR, rSr, and rsr accounted for 57.23% of the total, accounting for most cases.

Part D shows that the most common morphology after lowering the output in the NS-LBBP phase is rSR, which accounts for 32.89% of the total, followed by rsR, which occupies 19.74%. Similar morphology such as rSR, rsR, rSr, rsr, and rR’ accounted for 71.7% of the total, accounting for most cases.

Part E shows that the most common morphology in the S-LBBP phase is rsR, which accounts for 32.89% of the total, followed by rR’, which occupies 21.05%. The predominant morphology is with initial r waves, which includes variations like rSR, rsR, rSr, rsr, rR’, rs, rS, and R type, making up 96.71% of the total.

### 3.3. ECG vector in the V1 lead

The morphology of the phase transition process from LVSP to NS-LBBP to S-LBBP was analyzed, and most of the morphology changed in different phases. Whether the first ECG vector in the V1 lead is R/r or Q/q shows a significant difference in the pacing process. The probability of the appearance of R/r showed a substantial increase from LVSP to S-LBBP. Paired chi-square tests for the four stages showed significant differences in the initial r wave (*P* < 0.05) (Table 2).

**Table 2:** Paired chi-square test on whether the initial ECG vector is positive. Thenumber 0 means that the initial vector is negative, such as Q/q wave. 1 means that the initial vector is positive, such as R/r wave.

		R/r wave [NS-LBBP (low output)]		Total	*χ*2	p
		0	1			
R/r wave (LVSP)	0	60	61	121	22.076	<0.001
	1	1	30	31		
Total		61	91	152		
		R/r wave [NS-LBBP (lower output)]		Total	χ2	p
		0	1			
R/r wave [NS-LBBP (low output)]	0	34	27	61	42.797	<0.001
	1	7	84	91		
Total		41	111	152		
		R/r wave(S-LBBP)		Total	χ2	p
		0	1			
R/r wave [NS-LBBP (lower output)]	0	5	36	41	13.997	<0.001
	1	0	111	111		
Total		5	147	152		

The sensitivity of the initial r-wave for distinguishing LVSP from s-LBBP was 76.3%. The specificity could not be calculated because of the sample size.

**Figure 3:**
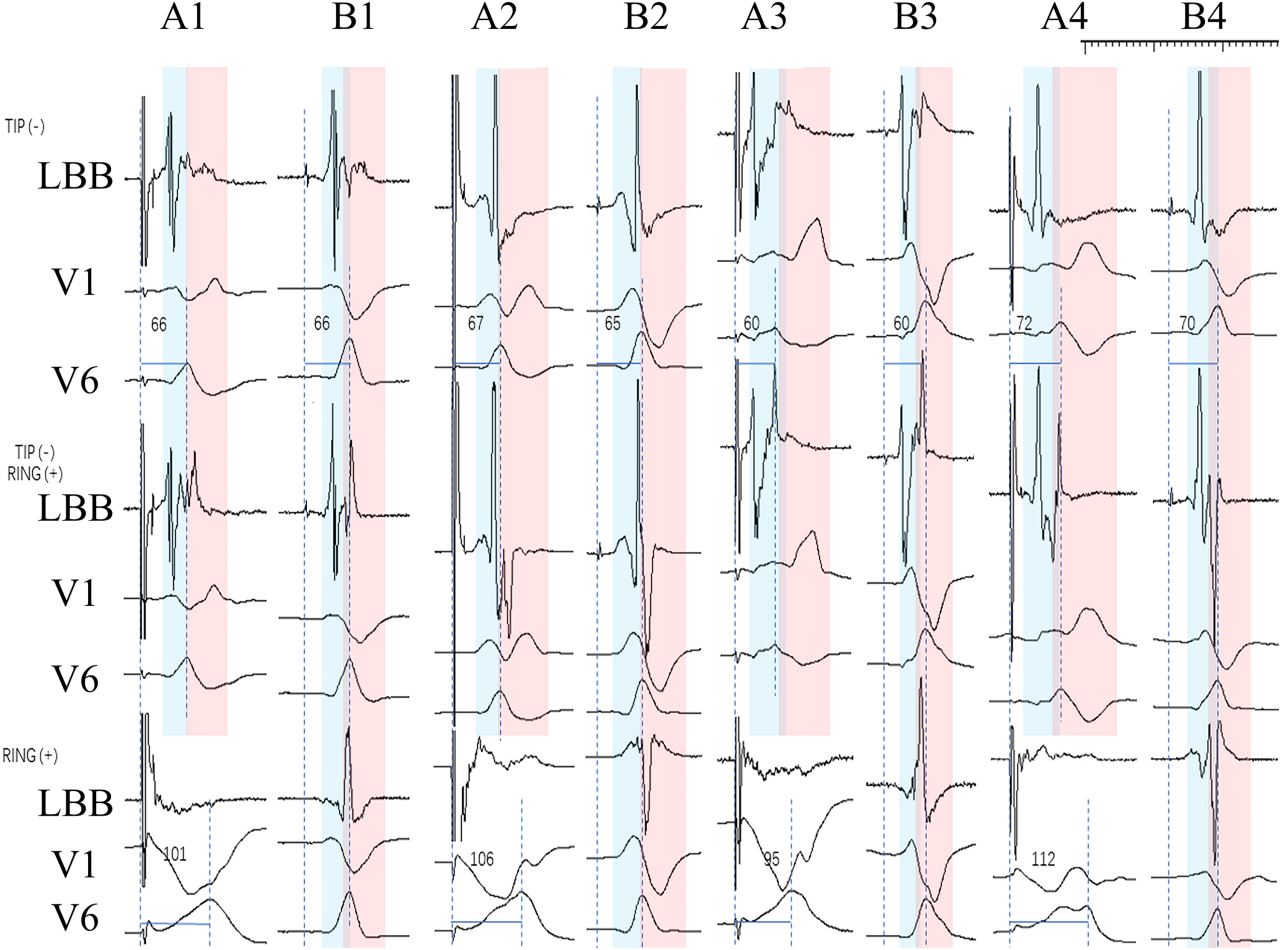
IEGM and ECG in the pacing and sense-only mode at the final test. Where A1-B1, A2-B2, A3-B3, A4-B4 are the same patient. TIP (-) in part B shows the ECG and IEGM in unipolar sense-only mode at the TIP end, RING (+) shows the ECG and IEGM in unipolar sense-only mode at the RING end, and TIP (-) RING (+) is the ECG and IEGM in bipolar sense-only mode. Part A (A1, A2, A3, A4) shows the ECG and IEGM in the pacing mode, including the TIP end on the left and the RING end on the right, as well as the bipolar mode. The ECG and IEGM in the TIP end is indicated by the blue bar, and the red bar indicates the ECG and IEGM in the RING end.

In Figure 3, Part A is the ECG and IEGM in the pacing mode, and Part B is the ECG and IEGM in the sense-only mode. TIP (-) in part B shows the ECG and IEGM in unipolar sense-only mode at the TIP end, RING (+) shows the ECG and IEGM in unipolar sense-only mode at the RING end, and TIP (-) RING (+) is the ECG and IEGM in bipolar sense-only mode. Similarly, part A is the ECG and IEGM in pacing mode. This means the graphs in part B are normal rhythms transmitted from the conduction bundle. The front of the ECG and IEGM in bipolar sense-only mode is derived from the standard conduction bundle at the TIP end, as shown in Figure 3 by the blue bar. The red bars show that the intra-cardiac electrogram in sense-only mode with RING end is similar to the latter part of the bipolar sense-only mode. This suggests that the first half of the electrogram in bipolar mode is caused by myocardial excitation sensed at the TIP end and the second half is caused by myocardial excitation sensed at the RING end. In the pacing mode, the morphological changes in the ECG and IEGM are similar, with the initial r wave in lead V1 appearing at the left TIP end, demonstrating that the V1 lead initial r waves are associated with excitation of the myocardium from normal left bundle branch.

### 3.4. RWPT in lead V1

The analysis of the RWPT in lead V1 is shown in Table 3. The results show that the RWPT was reduced after capturing the left bundle branch, from 113 ms to 108 ms (*P* < 0.001). During the NS-LBBP phase, a slight prolongation of the RWPT in V1 leads occurred during the lowering of the output from 108 ms to 110 ms (*P* < 0.001). And from the NS-LBBP phase to the S-LBBP phase, RWPT was significantly prolonged from 110 ms to 117 ms (*P* < 0.001) (Supplementary Figure 1).

**Table 3:** Comparison of RWPT in V1 leads in four phases. Data did not conform to a normal distribution and were finally expressed in the form of quartiles using the rank sum test, with *P* < 0.05 indicating a statistically significant difference in RWPT between the two stages.

RWPT-V1 (Item 1 vs. Item 2)	Median (P25, P75)		Median difference (item 1-item 2)	Z Value	*P* value
	Item 1	Item 2			
LVSP vs. NS-LBBP (low output)	113.000 (107.0,120.0)	108.000 (101.0,117.0)	4	5.544	<0.001
NS-LBBP (low output) vs. NS-LBBP (Lower output)	108.000 (101.0,117.0)	110.000 (103.0,119.0)	−2	3.792	<0.001
NS-LBBP (Lower output) vs. S-LBBP	110.000 (103.0,119.0)	117.000 (106.0,129.0)	−7	7.756	<0.001

## 4. Discussion

Limited by the long-term threshold instability of His bundle implantation, LBBP is currently a more stable alternative procedure. However, the exploration of LBBP is still limited.

This is the first article that summarizes the characteristics of ECG V1 lead morphology through a more significant number of S-LBBP cases. And it outlines how the essential V1 leads to change, exploring for the first time the enlightening effect of morphology changes and amplitude changes on left bundle branch capture.

Compared to the only published article on ECG morphology analysis (11), this study benefited from a continuous pacing and recording technique, which allowed for more accurate and real-time monitoring of events captured by the LBB.

In the S-LBBP, the more common morphology in lead V1 was rsR, which is inconsistent with the previous results (11). This article defines the ECG graphs as q-s-r with amplitude <0.5 mv; rR’ is defined as no negative wave, positive wave thwarted in front of the peak. If rSR, rsR, rSr, and rsr are uniformly defined as rR’ as in the previous study, then the most common S-LBBP is rR’ (12). And it occupies 94.73% of the majority in this study. In NS-LBBP, somewhat different from previously reported, the more common one was rSR, which was previously reported as the most common QR type (11). The most common morphology in the LVSP phase before LBB capture was QR.

The morphology of V1 correlates with how much of the myocardial component is excited and whether or not the LBB is captured. LVSP excites the myocardial cells and does not affect the conduction system. NS-LBBP (Low output) and NS-LBBP (Lower output) simultaneously excite the left myocardium and bundle branch. S-LBBP excites the conduction system, leading to a change in the depolarization vector of ventricular excitation, resulting in a difference in morphology.

On the other hand, our group has reported a shortening of RWPT with increasing screwing depth during the screwing of the electrode into the right ventricular septum to capture the LBB. Therefore, operators without continuous monitoring may be unable to detect the sudden shortening of RWPT in two adjacent heartbeats. This point chosen as the endpoint of the LBB capture may not be reasonable after most operators use the current method of intermittent measurement of RWPT. Therefore, there may be some bias in the previous morphology reports, which may be the main reason for the discrepancy in the study reports.

In 16 patients, when the pacing mode of LVSP changed to NS-LBBP, the QRS morphology transitioned from the QS pattern to the Qr or QR pattern with V6 RWPT shortened simultaneously. Usually, the subsequent change of the ECG vector from S to R or r is considered to be the arrival of the electrode in the left ventricular septum through the right ventricular septum, so it is presumed that the LBB of such patients may be close to the left of the middle intraventricular septal (IVS).

Except for five patients who showed qR pattern, most of the initial vectors of V1 leads in the S-LBBP showed positive, with r being the predominant one. This suggests that our ECG's initial positive vectors are possibly associated with left ventricular septal excitation through the LBB.

Among the two different output groups of NS-LBBP, the lower output group had a higher r or R occurrence rate in the initial vector than the low output group, with 71.7% (109/152) vs. 58.6% (89/152). The low and lower outputs affected only the range of direct myocardial excitation. The local LVS depolarization range is hypothesized to affect the V1 initial positive vector directly. Compared to the low output state at the beginning, the lower output reduced the extent to which the myocardium was directly agitated, reducing the incidence of the initial positive vector.

The initial r wave may be associated with myocardium activated by the downward transmission of the left bundle branch. In Figure 3, part B, where the blue bar shows the ECG and IEGM at the left TIP end, and the red bar shows the ECG and IEGM at the ring end by sense-only mode, a conclusion demonstrated by our previous research (13).

In clinical patient follow-up, the body surface ECG is more convenient than the intracardiac electrocardiogram marker. The initial r-wave in lead V1 may be a relative marker during the follow-up of patients with S-LBB capture.

In different phrases, the RWPT in V1 leads showed a change in the phase change of LVSP- NS-LBBP (low output)—NS-LBBP (lower output)- S-LBBP by first shortening and then lengthening. Previous studies have been conducted on the variation in V1 RWPT and V6RWPT duration during left bundle branch pacing. The V6 RWPT-V1 RWPT variation confirms LBB capture and distinguishes it from pure LVS myocardial capture (14). During the transition from NS-LBBP to S-LBBP, the V1RWPT lengthens significantly, while during the transition from LVSP to NS-LBBP, the V1RWPT shortens. Because the RWPT in V1 leads represents more of the excitation time of the right ventricle, the ventricular myocytes are still excited in the LVSP to NS-LBBP (low output) phase, and the increased output voltage has an accelerating effect on the conduction of electrical stimulation. This is in line with the results of the current study. The difference is that our study carried out the change in V1 RWPT during NS-LBBP (low output)—NS-LBBP (lower output). Although we do agree that a 1 ms time extension is not very meaningful for clinical practice.

After the transition from NS-LBBP (lower output) to S-LBBP, there is no direct excitation of the cardiomyocyte. The electrical activity is conducted exclusively through the conduction bundle. The electrical excitation propagates through the left bundle branch to the apex. The final electrical excitation is achieved through the left apical cardiomyocyte to the right ventricle. This process is similar to the right bundle branch block (RBBB) mechanism, increasing the conduction time and prolonging the right ventricular excitation time significantly.

**Figure 4:**
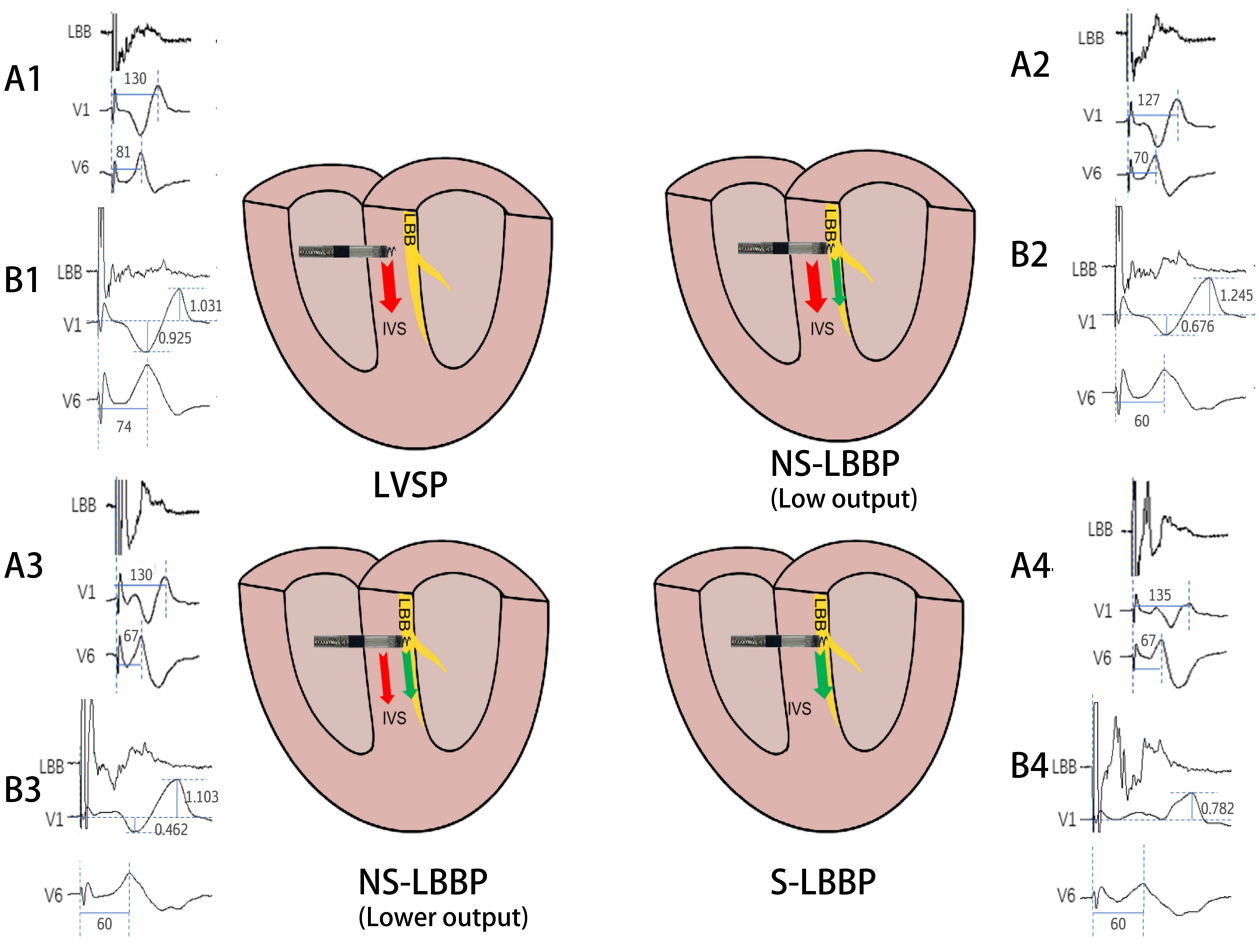
Schematic diagram of the conduction of electrical activity about the four nodes. The arrows indicate the direction of electrical activity conduction, red arrows are electrical activity conduction between cardiomyocytes and green arrows are electrical activity conduction of conduction bundles. A1, A2, A3, A4 and B1, B2, B3, B4 are ECG patterns of LBB, V1, V6 in two patients. A1: RWPT(ms) of V1 and V6 in LVSP phase. A2: RWPT(ms) of V1 and V6 in NS-LBBP (Low output) phase. A3: RWPT(ms) of V1 and V6 in NS-LBBP (lower output) phase. A4: RWPT(ms) of V1 and V6 in S-LBBP phase. B1: S- and R-wave amplitudes(mv) in lead V1 in LVSP phase. B2: Amplitude(mv) of S and R waves of V1 leads in NS-LBBP (Low output) stage. B3: Amplitude(mv) of S and R waves of V1 leads in NS-LBBP (Lower output) stage. B4: Amplitude(mv) of S and R waves of V1 leads in S-LBBP stage.

Overall, the findings of this study can provide more ideas about left bundle branch pacing and some insights into the different phases of ECG (Figure 4). In areas where continuous monitoring cannot be used, this may provide the operator with more information during the procedure.

## 5. Limitation

First, the number of cases in this study is still small, which may impact the study's conclusions, and clinicians need to interpret the findings with caution. Secondly, V1 leads of body surface ECG can only provide some essential ideas for pacing procedures. There are some baseline instabilities in the waveform judgment and measurements, which may have an effect on the results. And these findings apply to normal hearts and those with cardiomyopathy do not fully apply.

## 6. Conclusion

This study supports the use of morphology changes in V1 leads for the adjunctive determination of the different phases of left bundle branch pacing, especially in the intraoperative context of intermittent morphology monitoring. The initial r-wave in lead V1 may be a relatively good marker during the follow-up of patients with left bundle branch capturing.

## Data Availability

The original contributions presented in the study are included in the article/**Supplementary Material**, further inquiries can be directed to the corresponding author.
