# Corrigendum: Guidance on left bundle branch pacing using continuous pacing technique and changes in lead V1 characteristics under real-time monitoring

**DOI:** 10.3389/fcvm.2023.1326956

**Published:** 2023-11-29

**Authors:** Nan Zheng, Longfu Jiang, Jiabo Shen, Jinyan Zhong

**Affiliations:** Department of Cardiovascular Medicine, Ningbo NO.2 Hospital, Ningbo, China

**Keywords:** left bundle branch pacing, left bundle branch capture, physiological pacing, left ventricular septal capture, electrocardiogram

A Corrigendum on Guidance on left bundle branch pacing using continuous pacing technique and changes in lead V1 characteristics under real-time monitoring By Zheng N, Jiang L, Shen J, Zhong J (2023). Front. Cardiovasc. Med. 10. doi. 10.3389/fcvm.2023.1195509

**Error in Figure/Table**

In the published article, there was an error in Figure 4 as published. There are some confusing marks in the diagram. The corrected Figure 4 and its caption appear below.

**Figure 4:**
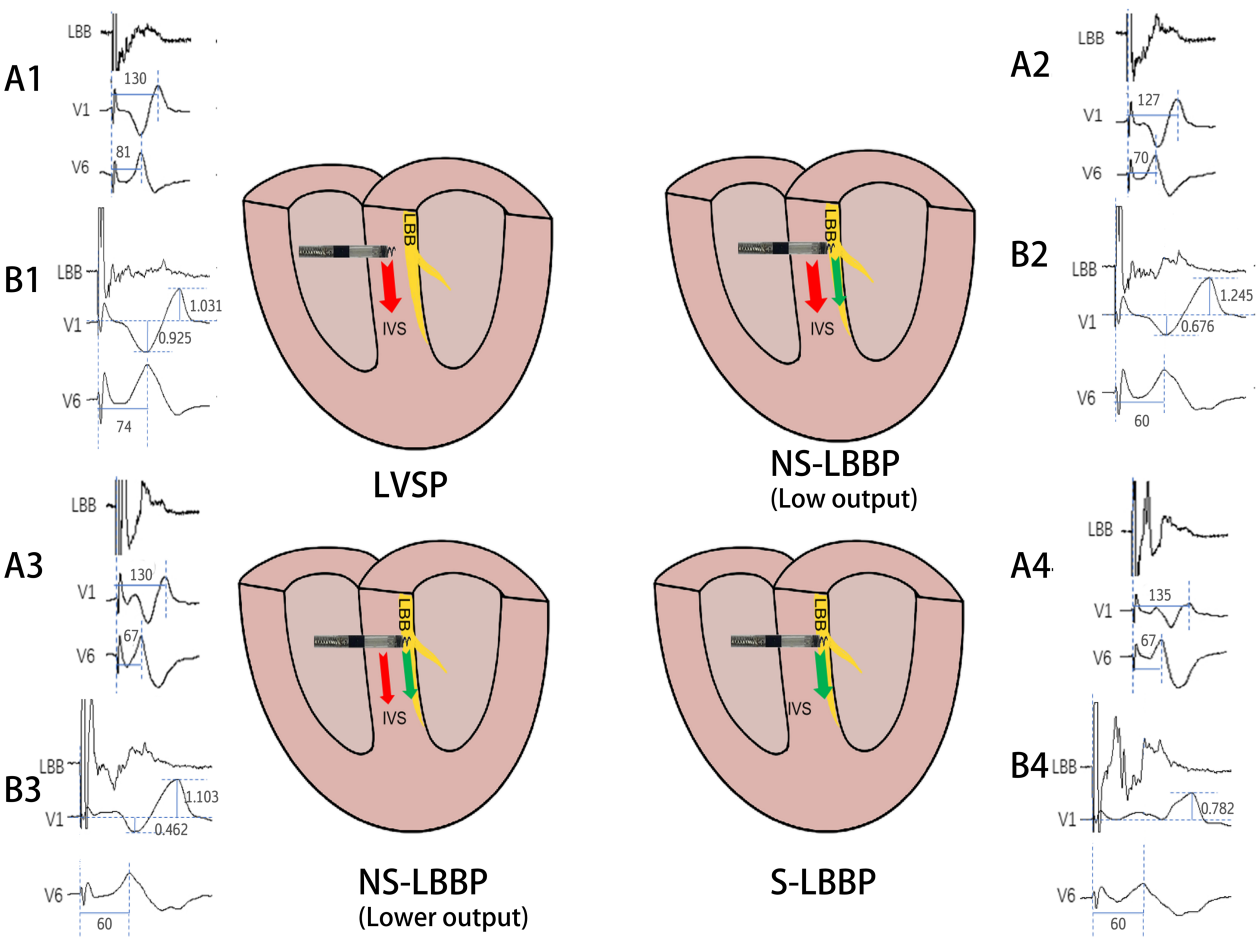
Schematic diagram of the conduction of electrical activity about the four nodes. The arrows indicate the direction of electrical activity conduction, red arrows are electrical activity conduction between cardiomyocytes and green arrows are electrical activity conduction of conduction bundles. A1, A2, A3, A4 and B1, B2, B3, B4 are ECG patterns of LBB, V1, V6 in two patients. A1: RWPT(ms) of V1 and V6 in LVSP phase. A2: RWPT(ms) of V1 and V6 in NS-LBBP (Low output) phase. A3: RWPT(ms) of V1 and V6 in NS-LBBP (lower output) phase. A4: RWPT(ms) of V1 and V6 in S-LBBP phase. B1: S- and R-wave amplitudes(mv) in lead V1 in LVSP phase. B2: Amplitude(mv) of S and R waves of V1 leads in NS-LBBP (Low output) stage. B3: Amplitude(mv) of S and R waves of V1 leads in NS-LBBP (Lower output) stage. B4: Amplitude(mv) of S and R waves of V1 leads in S-LBBP stage.

The authors apologize for this error and state that this does not change the scientific conclusions of the article in any way. The original article has been updated.

