# Supplementary material for: Guidance on left bundle branch pacing using continuous pacing technique and changes in lead V1 characteristics under real-time monitoring
Source: Front Cardiovasc Med. 2023 Sep 29;10:1195509. doi: 10.3389/fcvm.2023.1195509 (PMC10570442; doi:10.3389/fcvm.2023.1195509)
Supplement: Supplementary file 1 [file Datasheet1.docx]

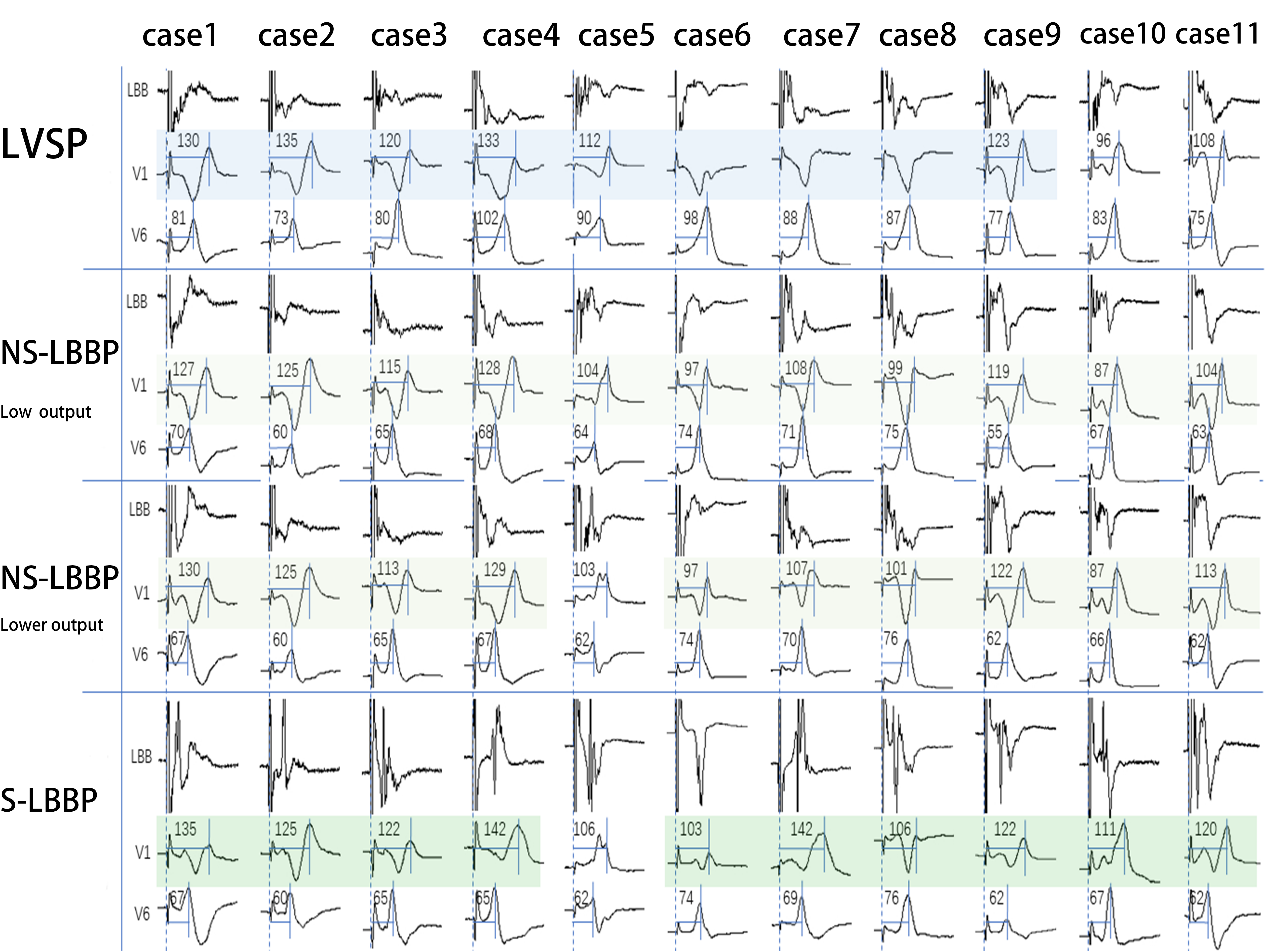


Supplementary Figure 1: RWPT of V1 and V6 in the four nodes of LBBP. Cases 1-11 are some of the patients with selective left bundle branch pacing. LVSP to NS-LBBP (Low output) phase, RWPT in V6 leads was significantly shortened, and subsequent NS-LBBP (Lower output) and S-LBBP showed a stable state. The RWPT time in V1 leads showed by first shortening and then lengthening.
